# Illness Severity in Psychotic Disorders Amplifies Anterior Insula’s Sensitivity to Unreciprocated Smiles

**DOI:** 10.5334/cpsy.142

**Published:** 2025-12-30

**Authors:** Jayson Jeganathan, Megan E. J. Campbell, Renate Thienel, Nikitas C. Koussis, Bryan Paton, Katharina V. Wellstein, Michael Breakspear

**Affiliations:** 1 School of Psychological Sciences, College of Engineering, Science and the Environment, University of Newcastle, Newcastle, NSW, Australia; 2 Hunter Medical Research Institute, Newcastle, NSW, Australia; 3 School of Health and Rehabilitation Science, The University of Queensland, Brisbane, QLD, Australia; 4 School of Medicine and Public Health, College of Medicine, Health and Wellbeing, University of Newcastle, Newcastle, NSW, Australia; 5 Mark Hughes Foundation Centre for Brain Cancer Research, University of Newcastle, NSW, Australia

**Keywords:** schizophrenia, psychosis, fMRI, magnetic resonance imaging, facial expression, emotion, negative symptoms

## Abstract

When we smile, we expect that others will smile back. When one’s smile is not reciprocated, these expectations are violated, producing prediction error signals in the brain. Prediction error signals may be experienced as aversive, disincentivizing smiling. Social smiling is impaired in psychotic disorders suggesting increased sensitivity to unreciprocated smiles.

We developed the Incongruent Facial Emotion task to probe responses to unreciprocated smiles. Healthy controls and persons with schizophrenia or schizoaffective disorder voluntarily smiled, after which they viewed a stimulus face with a happy or angry expression. Brain activations were quantified with functional magnetic resonance imaging.

Greater illness severity was associated with reduced smile amplitude. Across both groups, viewing an incongruent stimulus after initiating a smile activated the bilateral anterior insulae and right supplementary motor cortex. Brain activations in the left middle occipital and left superior frontal gyri were greater in the clinical group. The anterior insula response to incongruent facial reactions was significantly greater in more severely ill clinical participants. Dynamic causal modelling suggests that incongruent stimuli reduce tonic self-inhibition in the anterior insula, and that this disinhibition is enhanced by illness severity.

The results suggest that the anterior insula processes affective prediction errors and sends feedback to supplementary motor areas to alter behavioural responses. The underlying brain circuits are enhanced in clinical participants with severe illness, suggesting new avenues to understand affective blunting in psychotic disorders.

## Introduction

Diminished facial expressivity and impaired social reciprocity are core features of primary psychotic disorders. These symptoms have a substantial impact on overall illness severity, persist even after active psychosis remits, and are relatively resistant to pharmacological interventions (Fenton & McGlashan, 1991; Galderisi et al., 2018; Piskulic et al., 2012; Sheitman & Lieberman, 1998). They are further exacerbated by secondary factors like environmental deprivation and social exclusion (Foussias et al., 2014; Malaspina et al., 2014). These symptoms are generally measured with rating scales such as the Positive and Negative Symptom Scale (Kay et al., 1987) or Brief Negative Symptom Scale (Kirkpatrick et al., 2011), which are inherently subjective and hence less reliable (Weigel et al., 2024). Quantitative measurements of facial movements with electromyography or facial video in psychotic disorders have found flatter facial expressions (Berenbaum & Oltmanns, 1992; Hamm et al., 2011; Tron et al., 2016), increased frequency of inappropriate expressions (Alvino et al., 2007; Wang et al., 2008), and reduced smile-related activity (Kohler et al., 2008). One theory proposes that negative symptoms arise from impaired dopamine reward signalling (Deserno et al., 2017). However, this theory does not have social specificity, and focuses on amotivation rather than emotional expressivity. For example, reward discounting deficits are associated with apathy but not with diminished expression (Hartmann et al., 2015). There is an urgent need to understand the neural basis of social-emotional reciprocity in health, and how this is disrupted in schizophrenia and schizoaffective disorder.

Another theory extends the framework of the dysregulated dopaminergic neurotransmission in schizophrenia, proposing that negative symptoms are caused by impaired striatal dopaminergic signalling of positive reward prediction errors. This leads to a bias towards learning the negative value of action, and a failure of flexible behavioural adaptation (Deserno et al., 2017). The theory is supported by performance on probabilistic reward tasks, striatal neuroimaging abnormalities, and the non-response of negative symptoms to anti-dopaminergic treatments (Strauss et al., 2014). However, this theory does not have social specificity. It focuses on avolition-apathy rather than on emotional expressivity, to the extent that some studies have excluded the ‘restricted affect’ item of the SANS questionnaire (Gold et al., 2012), while others have found that effortful discounting deficits are associated with apathy but not diminished expression (Hartmann et al., 2015). Moreover, functional neuroimaging studies of affective blunting implicate a host of extrastriatal regions (Benoit et al., 2012; Stip et al., 2005). Finally, it is unclear how intact signalling of punishment with negative reward prediction errors is consistent with impairments in expressing negative emotions in schizophrenia (Kohler et al., 2008).

The brain circuits implicated in facial expressions have been well studied in health. Performing voluntary facial movements activates the primary motor cortex, premotor and supplementary motor cortices, putamen, thalamus, and insula (Krippl et al., 2015). Imitating the facial expression of others activates regions collectively framed as the *human mirror system* – including inferior frontal gyrus, inferior parietal lobule, premotor areas, insula and amygdala (Iacoboni et al., 1999; Pohl et al., 2013). One study found that individuals with schizophrenia have reduced activity in dorsal frontal regions during facial imitation(Lee et al., 2014), but this finding has not been replicated (Horan et al., 2014).

Smiling is driven not just by imitation but occurs broadly in social encounters. Social smiling can be understood through the predictive processing framework. The tenets of this framework are as follows. The brain continually predicts future sensory inputs. Violated predictions produce *surprise* signals in the brain, also known as prediction error (PE) signals. Predictive coding suggests that the brain strives to minimize PEs in order to accurately represent the external world. Viable strategies to minimize PEs include updating one’s predictions, issuing motor outputs to change the environment, or changing one’s behaviour (K. Friston, 2005; K. J. Friston et al., 2014).

In the case of facial expressions, smiling can be seen as being conditioned upon a prediction that others will most likely respond positively or smile back. Unreciprocated smiles are usually surprising and lead to a PE signal in the brain. One potential strategy to minimize these long term PEs may be to avoid spontaneous smiling in social contexts (Jeganathan & Breakspear, 2021). Empirical evidence shows that smiling is amplified when people expect the smile to be reciprocated (Heerey & Crossley, 2013), while ongoing social engagement is avoided when others respond with neutral or negative expressions (Marsh et al., 2005).

Smiling is impaired in those with schizophrenia and schizoaffective disorder, particularly those with more severe illness (Galderisi et al., 2018). Blunting of positive affect suggests a disruption in the brain circuits underlying affective PEs. To test this hypothesis, we developed a customized fMRI task where participants smiled while viewing a stimulus face that appeared to react with either a happy or angry expression. Since persistent PE signals from unreciprocated smiles disincentivize smile behaviour and social engagement, we hypothesized that the resulting PE signals would be different in individuals with psychotic disorders, and particularly in clinical participants with greater illness severity. Increased sensitivity to unreciprocated smiles could plausibly lead to impaired sociality in those with severe psychotic illness. If this were the case, severely ill individuals may attenuate their smiles to limit prediction errors arising in social interactions.

## Methods

### Participants

The study cohort comprised 40 clinical participants diagnosed with psychotic disorder and 35 healthy controls. All participants were aged 18–50 years. Clinical participants fulfilled DSM-5 criteria for psychotic disorder (schizophrenia or schizoaffective disorder), while control participants disavowed a history of severe mental illness (see Supplementary Methods 1 for more details).

### Clinical interview

Cognitive function was assessed with the Wechsler Abbreviated Scale of Intelligence (WASI-II). In the clinical cohort, illness severity was measured with the Clinical Global Impression (CGI), a measure of overall illness severity. A single measure was used to reduce the impact of multiple testing on statistical power. CGI has been found to be comparable to composite measures derived from multi-dimensional scales for identifying symptom change in psychotic disorders (Pinna et al., 2015; Samara et al., 2014). The clinical cohort also completed the following scales: Social and Occupational Functioning Assessment Scale (SOFAS) (Morosini et al., 2000), Positive and Negative Symptom Scale (PANSS) (Kay et al., 1987), Hamilton Depression Rating Scale (HAM-D) (Hamilton, 1960), and Simpson-Angus Scale (SAS) (Hawley et al., 2003) for extrapyramidal side effects. The information for all scales and for the overall diagnosis was obtained from a combination of semi-structured clinical interview, and review of inpatient discharge summaries and outpatient psychiatric notes completed by one of the authors (J.J. a senior psychiatry registrar). Where the diagnosis remained uncertain, consensus was achieved by discussion between two coauthors, J.J. and M.B. (board registered psychiatrist).

### Incongruent Facial Emotion task

**Figure 1:**
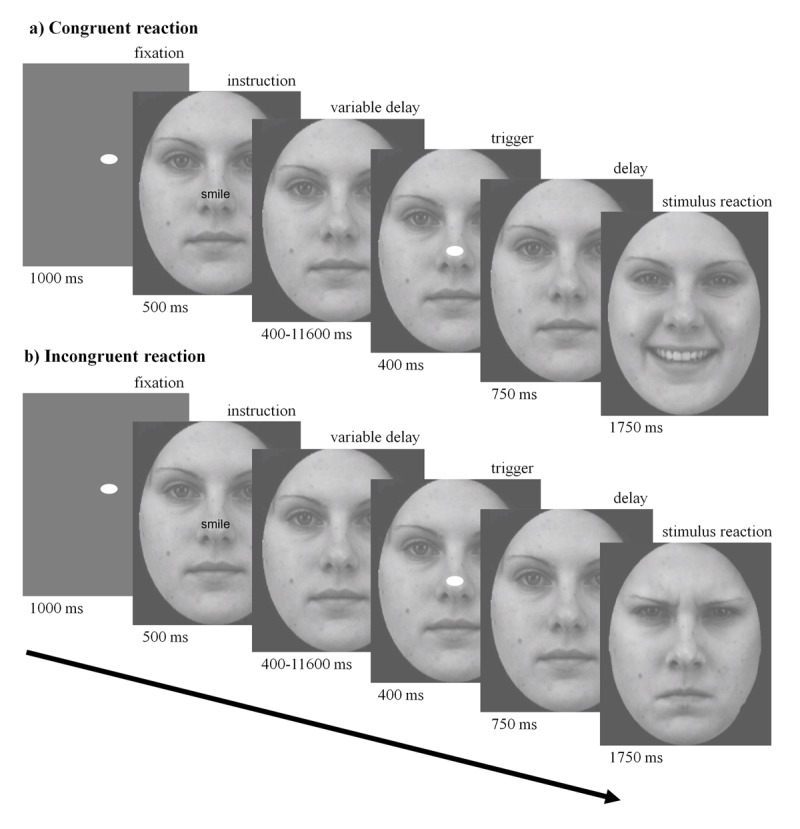
Trial structure of the Incongruent Facial Emotion task. At the beginning of the task, the following instructions were displayed: “When instructed, either frown or smile. The face on the screen will then make a facial expression”. Each trial had the following structure. Participants fixated on a fixation dot at the centre of the screen (1000 ms). An instruction to smile or frown appeared at the centre of the screen (500 ms). Participants were told to remember the instruction but not to smile or frown yet. There was a variable delay (400–11,600 ms) between this instruction and the trigger to start smiling or frowning. The variable delay allowed the fMRI activity associated with making facial expressions to be dissociated from the fMRI activity to viewing the instruction. Delays were calculated using the optseq2 tool to optimize statistical power (Dale, 1999). The trigger was the reappearance of a fixation dot lasting 400 ms. Participants were instructed to smile or frown at the appearance of the trigger. Participants held the expression until the face was replaced by a fixation dot indicating the end of the trial. After an additional delay (750 ms), participants viewed the stimulus’ reaction (1750 ms), a gradual transformation of the neutral face to either a happy (panel a, condition *Hh*) or angry (panel b, condition *Ha*) expression (35 frames × 50 ms per frame). At the conclusion of the trial (the start of the next fixation period), participants were asked to return to a neutral facial expression.

We developed a task to identify brain regions that activate when one perceives incongruent facial expressions. The task comprised 80 consecutive trials with mean trial duration of 6.4s and total task time of 8 min 52 sec. Each trial corresponded to one of four conditions: *Hh, Ha, Ah*, or *Aa*. Participants were instructed either to smile (H) or to produce an angry frown (A) while viewing a static neutral face. After a short interval, the stimulus face transitioned to a dynamic facial expression, either happy (h) or angry (a). For example, in *Ha* trials, a participant smiles, but the stimulus reacts with an angry expression. *Hh* and *Aa* trials corresponded to a congruent stimulus, while *Ha* and *Ah* trials corresponded to an incongruent stimulus. There were 20 presentations of each of the four trial types (Figure 1). Trials were presented in pseudorandomised order to prevent anticipation effects while optimising statistical power. Face stimuli were obtained from the Karolinska Directed Emotional Faces dataset (Lundqvist et al., 1998) (Supplementary Methods 2).

Participants completed a practice run (20 trials) of the Incongruent Facial Emotion task, followed by two task runs. The first task run was conducted outside the scanner and included simultaneous video recording of facial expressions. The second task run had fMRI acquisition but no facial video, because fitting a suitable camera within the scanner bore is not practicable (see Supplementary Methods 3 for further details of video recording and stimulus presentation).

### Analysis of facial expression data

Participants’ facial expressions in the video footage were quantified with the Facial Action Coding System (FACS) (Ekman et al., 1978). This system decomposes facial expressions into activations of a set of action units (AUs), each of which corresponds to a facial muscle. For instance, smiling is most associated with AU12 “Lip Corner Puller”, which corresponds to contraction of the zygomatic major muscle (Ekman et al., 1978). OpenFace software was used to extract facial landmarks and frame-by-frame activations for 16 AUs. This software employs automated analysis but has high concordance with manual ratings (Baltrusaitis et al., 2018). Smile amplitude, smile and frown onset latency were estimated from these time series (Supplementary Methods 4). Quality control for facial expression data comprised the following. Participants whose facial landmarks were not detectable in more than 10% of webcam frames were excluded (2 excluded). Participants whose smile onset latency was not estimable in more than 30% of trials were excluded (7 excluded).

### Statistical analysis

Continuous variables were tested for normality with the Shapiro-Wilk test. Group differences were tested with two-sample unpaired t-test (unequal variance) for normally distributed outcomes, or the Mann-Whitney U test for non-normal outcomes. Correlations between outcomes were calculated using Pearson’s *r* for normally distributed outcomes, and Spearman’s rho for non-normal outcomes. The Benjamini-Hochberg procedure was used for false discovery rate correction (Benjamini & Hochberg, 1995).

### fMRI data analysis

fMRI data were acquired during the Incongruent Facial Emotion task (Supplementary Methods 5). fMRIPrep version 23.0.0 was used for preprocessing (Supplementary Methods 6). Four participants with failed anatomical segmentation were excluded. fMRI data quality for each participant was assessed using fMRIPrep’s built-in visual quality assessment reports. The general linear model was implemented in SPM version 12 v7771, with regressors for each of the task condition (*Hh, Ha, Ah, Aa*). Second level general linear model with random effects analysis was used to find significant task effects and group differences (see Supplementary Methods 7 for details). Three methods were used to minimize head motion artifacts. First, task condition regressors had value 1 during the stimulus reaction phase, when participants were asked to refrain from changing their facial expression, and value 0 during the trigger, delay and fixation phases, when participants would smile, frown, or return to a neutral expression. Second, residual motion artifacts were automatically identified and removed with ICA-AROMA (Pruim et al., 2015). Third, head movement time series were estimated from BOLD data (Supplementary Methods 6) and included as nuisance covariates in the design matrix (Supplementary Methods 7). Although we selected CGI as our primary illness severity measure, we also examined associations with PANSS subscales (Supplementary Table 5) to characterize symptom-brain relationships.

### Dynamic causal modelling

Task-associated activation in a specific brain region can be caused by direct task effects on that region, task effects relayed from upstream regions, or by modulation of inter-areal effective connectivity between upstream regions and the target region (K. J. Friston et al., 2003). After significant clusters corresponding to task effects were identified, dynamic causal modelling (DCM version 12.5) was used to test plausible hypotheses regarding the upstream causes of identified activations. We tested whether the incongruent frowning stimuli amplify a region’s activity directly, or by modulating incoming connectivity from other brain regions. Different models were each specified by parameters corresponding to system inputs (facial stimuli), the intrinsic network connections, and modulatory *bilinear* parameters. Modulatory parameters explain how network connections are modulated by task manipulations, in our case whether the stimulus facial reaction was congruent or incongruent. The best models were identified with parametric empirical Bayes (Supplementary Methods 8).

## Results

### Demographics

75 participants (40 clinical and 35 control) were recruited. 9 participants (6 clinical) were excluded based on lower quality facial expression data in the behavioural suite task run (Supplementary Methods 4). An additional 6 participants (2 clinical) were excluded due to MRI quality control. Of these, one participant was excluded due to missing field maps, one participant was excluded due to failed heartrate recording, and four participants were excluded due to failed brain tissue segmentation which erroneously included non-brain tissue such as eyes or facial sinuses within the brain mask.

The remaining 60 participants were included in the analyses (32 clinical participants). Within the clinical group, we did not find significant differences between the included and excluded participants in terms of PANSS positive symptoms (*U* = 161, *p* = 0.277), PANSS negative symptoms (*t*(38) = 0.252, *p* = 0.802), or CGI (*U* = 96, *p* = 0.275). Within the included 60 participants, there were no significant differences between the clinical and control groups in sex or education. Mean age and IQ were lower in the clinical group compared to the healthy participants (age: Mann-Whitney U test, *U* = 222, *p* < 0.001; IQ: two-sample t-test, *t*(58) = –2.577, *p* = 0.013) (Supplementary Table 1). In the clinical group, 30 participants identified as Caucasian, one as East Asian and one as African. All clinical participants were prescribed antipsychotic medication, with a mean chlorpromazine equivalent antipsychotic dose of 584 mg (SD 381 mg). Supplementary Table 2 provides mean clinical ratings in the clinical cohort. Analysis of covariance (ANCOVA) was used to test for group differences in behavioural responses, with age as a nuisance covariate. Age was also included as a covariate in tests of group differences in neural responses.

### Facial responses

**Figure 2:**
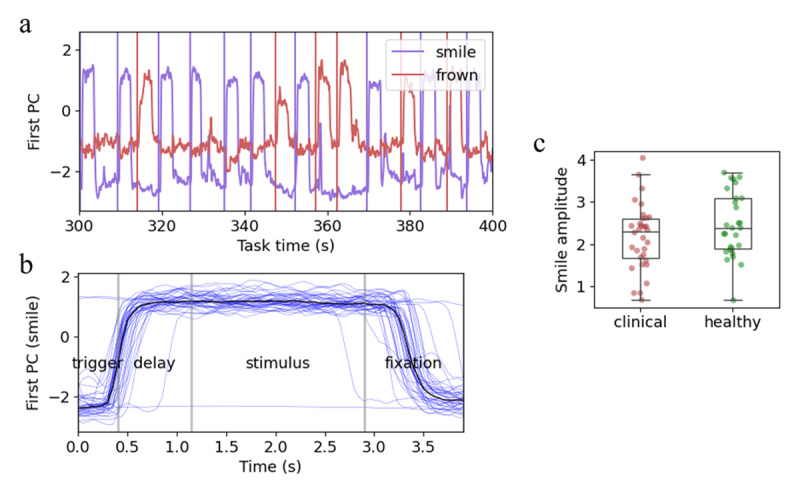
Analysis of single-trial voluntary facial expression time series in a sample participant (participant 020). **a)** Exemplar time series for the first principal components (PC) of smile and frown responses. PCs were estimated in each participant separately. The first PC corresponds to the unique combination of facial action units that are most activated in that participant. Vertical lines show triggers indicating that the participant should smile (purple) or frown (red). **b)** A single participant’s trial-wise facial responses to smile-instruction trials. Time zero represents the trial’s trigger. Vertical grey lines demarcate trial segments. Black line represents the median response across all trials. **c)** Smile amplitude in the clinical and control groups. Each dot represents a single participant. Boxplots show data quartiles.

Facial video footage from the Incongruent Facial Emotion task was used to extract trial-specific facial responses (Figure 2b). Voluntary smile amplitude and smile/frown latency were estimated from these time series. There were no significant group differences in voluntary posed smile amplitude (F(1,57) = 1.978, *p =* 0.165) (Figure 2c), smile latency (F(1,57) = 0.678, *p* = 0.414), or frown latency (F(1,57) = 0.887, *p* = 0.350). Within the clinical cohort, high illness severity (CGI) was significantly associated with reduced smile amplitude (Spearman’s ρ = –0.360, *p* = 0.043) and increased frown latency (ρ = 0.464, *p* = 0.007) but not with smile latency (ρ = 0.013, *p* = 0.944). There were no significant associations between PANSS positive or negative symptom subscales, and these voluntary facial response metrics.

### Neural activations: main effects and interactions

Overall task responses to the Incongruent Facial Emotion task occurred in bilateral visual areas, fusiform gyrus, pre- and post-central gyri, and parietal association areas (all cluster-level FDR_p_ < 0.05) (Supplementary Table 3). Significant main effects of participant action (smiling vs. frowning) occurred in early visual areas, precuneus, inferior temporal gyrus, and superior parietal lobule. Significant main effects of stimulus emotion occurred in parahippocampal gyri, lingual gyrus, fusiform gyrus, inferior temporal gyrus, and right inferior frontal gyrus. Compared to incongruent stimuli, congruent trials where stimuli matched the participant’s posed expression activated the bilateral putamen, caudate, right superior parietal lobule, right supramarginal and angular gyri, fusiform cortex, and early visual areas (Supplementary Table 3, Supplementary Figure 2). There were no significant clusters corresponding to incongruent stimuli. We then tested for a three-way interaction of group, participant action, and stimulus. In the postcentral gyri bilaterally, the Incongruent>Congruent response was greater in clinical participants than in the control group (Supplementary Table 5).

### Smile-specific neural activations

**Figure 3:**
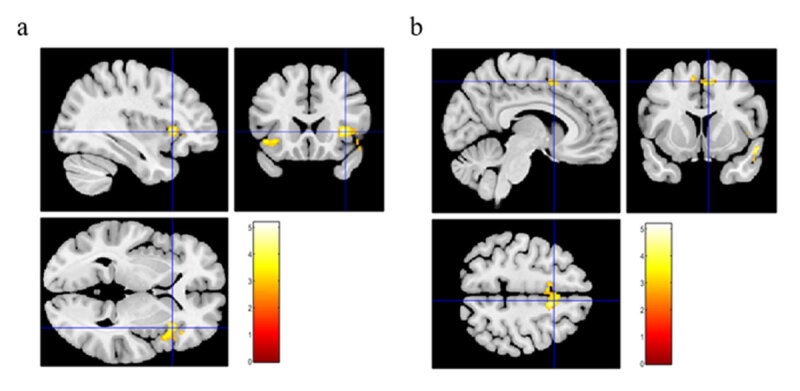
Clusters with significant activity when participants’ voluntary smile was followed by an incongruent angry stimulus. Left: right anterior insula, MNI coordinates (37.0, 19.2, 1.0). Right: right supplementary motor cortex, MNI coordinates (5.2, 7.5, 54.0).

To test our central hypotheses – namely the role of prediction error (PE) signals arising from unreciprocated smiling – we next focused on brain activity following incongruent reactions to smile trials. We therefore tested for brain regions whose activity increased when the participant’s smile was followed by an incongruent frowning stimulus (Ha>Hh contrast). Significant clusters were found in bilateral anterior insulae (AI), right inferior frontal gyrus, and right supplementary motor cortex (SMC) (Supplementary Table 4, Figure 3).

When viewing incongruent stimuli after smiling, clinical participants had greater activity in left middle occipital and left superior frontal gyri (Supplementary Table 5). Within the clinical group, participants with greater overall illness severity (CGI) exhibited increased activations to incongruent stimuli while smiling in several regions including the ventral midbrain, left middle frontal gyrus, right AI, and bilateral SMC. Notably, the AI and SMC were not only activated by unreciprocated smiles in the larger cohort (controls + clinical), but activity in these regions was also boosted by illness severity in the clinical group. In addition, clinical participants that exhibited greater smile amplitude during the Incongruent Facial Emotion task showed smaller activations in the right AI, right postcentral gyrus, and right amygdala (Supplementary Table 5). Equivalently, those with reduced smile amplitude had greater activations to incongruent stimuli. Clinical participants with greater PANSS positive symptom score had increased activations to incongruent stimuli in the cerebellar vermis, left angular gyrus, left medial precentral gyrus, and left fusiform gyrus. No associations were seen with PANSS negative symptoms. Finally, higher antipsychotic doses were correlated with response to incongruent stimuli in the right precentral gyrus (Supplementary Table 5).

### Dynamic causal modelling

**Figure 4:**
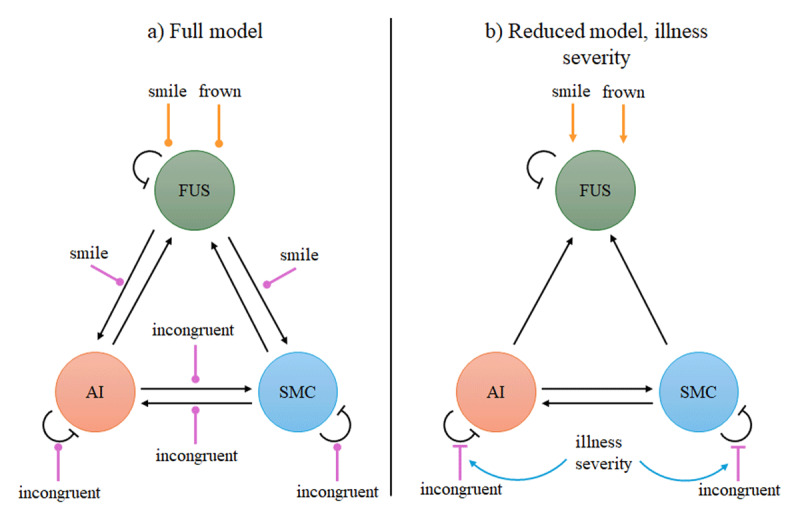
Dynamic causal models with inter-areal connectivity (black arrows), direct task inputs (orange), and bilinear modulation of inter-areal connectivity by task (purple). Blue arrows indicate the effect of illness severity on bilinear modulation terms. Regular arrowheads indicate excitatory connections. Flat arrowheads indicate inhibitory connections. Network nodes comprised right fusiform (FUS), right anterior insula (AI), and right supplementary motor cortex (SMC). **a)** Full model. Bilinear connections were tested for significance. Circular arrowheads indicate that prior to model reduction, the valence of connections (excitatory, inhibitory) is unknown. **b)** Reduced model with illness severity (CGI) as a covariate. Participants with greater illness severity had greater disinhibition of AI and SMC by incongruent stimuli.

Two questions arise from these findings. First, what are the neural processes underlying brain activations to unreciprocated smiles across all participants; and second, what processes underlie the increased sensitivity to unreciprocated smiles in clinical participants with more severe illness. Brain activations to unreciprocated smiles (our central hypothesis) were seen only in the right AI, right IFG, and SMC. We employed dynamic causal modelling (DCM) to test different plausible hypotheses that could explain activations in the right AI and the right SMC. These nodes were selected for the following reasons. Previous work has implicated the AI in incongruent affective reactions (Benuzzi et al., 2023), and in the integration of sensory inputs to influence emotional state (Paulus & Stein, 2006). Explaining activity in the SMC, a pre-motor area, was of substantial interest because we hypothesized that greater prediction errors impair facial motor activity. The DCM’s focus on AI and SMC was also motivated by the finding that activity in these regions was greater in more severely ill participants. In the DCM, these two network nodes were directly or indirectly modulated by the stimulus face, and by the participant’s facial motor response. Since the task primarily involved processing the stimulus face, the right fusiform cortex (FUS), a face processing region (Kanwisher et al., 1997), was added as a third network node for processing visual face input. Activity time series in the three network nodes were extracted (for more detail see Supplementary Methods 8). The base network model was specified as follows. Each of the three regions was modelled to receive input from every other region, and self-inhibitory feedback. The FUS node was modelled to receive direct task input from the smile and frown regressors. Effective connectivity from FUS to the AI and SMC were specified to be modulated by the smile regressor, providing further means by which participant smiling could modulate responses in both regions (Figure 4a). The incongruent stimulus regressor was the difference between Ha trials (smile instruction followed by angry stimulus reaction) and Hh trials (smile instruction followed by happy stimulus reaction) (for more detail see Supplementary Methods 8). The model represented two plausible hypotheses to account for higher AI activation in response to viewing incongruent, frowning reactions while smiling. The incongruent stimulus regressor was modelled to either modulate the AI’s self-inhibition or incoming connectivity from the SMC. Similarly, two hypotheses were considered for the SMC’s response, with either potential modulation of its self-inhibition or of incoming connectivity from the AI (Figure 4a). These represent alternative specifications of the B-matrix, which captures how experimental conditions modulate network connectivity.

We found strong evidence (posterior probability *Pp* > 0.95) that FUS activity is increased when viewing face stimuli while smiling (expected posterior E_p_ = 0.185 Hz, *Pp* > 0.99) or frowning (expected posterior E_p_ = 0.139 Hz, *Pp* > 0.99). Activity in AI and SMC were mutually reinforcing (AI to SMC, E_p_ = 0.239 Hz, *Pp* > 0.99; SMC to AI, E_p_ = 0.360 Hz, *Pp* > 0.99), and fed back to the FUS (AI to FUS E_p_ = 0.144 Hz, *Pp* > 0.99; SMC to AI, E_p_ = 0.180 Hz, *Pp* > 0.99) (Figure 4b, Supplementary Figure 3a).

We found strong evidence that incongruent frowning stimuli (Ha trials: participant smile followed by angry face) reduces tonic inhibition in both the right AI (expected posterior E_p_ = –1.511 Hz, *Pp* > 0.99) and the SMC (E_p_ = –1.473, *Pp* > 0.99). The negative expected posterior values indicate relaxation of each region’s inhibitory self-connectivity (Figure 4b, Supplementary Figure 3b). This represents reduced damping in the system’s dynamics and is referred to as ‘disinhibition’ in the rest of the paper.

To address the second question, we asked whether illness severity (CGI scale) increases activation of the right AI by modulating the disinhibition of AI, SMC, or both. Within the clinical cohort, we thus tested whether the influence of incongruent stimuli on effective connectivity is modulated by illness severity. In participants with greater overall symptom burden, incongruent stimuli were associated with greater disinhibition of right AI (E_p_ = –0.903 Hz, *Pp* > 0.99) and SMC (E_p_ = –0.747 Hz, *Pp* > 0.99) (Figure 4b, Supplementary Figure 3c). That is, illness severity amplifies how strongly incongruent social feedback perturbs the network’s dynamics.

## Discussion

Across all participants, viewing incongruent reactions while smiling activated the anterior insula (AI) and supplementary motor cortex (SMC). Using DCM, we found that incongruent stimuli result in amplified BOLD activity in the AI and SMC by decreasing their tonic self-inhibition. These findings align nicely with our recent theoretical work postulating that smile behaviour is driven by expectations that others will smile back, and that unexpected affective responses from another person lead to prediction errors (PE) (Jeganathan & Breakspear, 2021). Activity in the AI and SMC in response to unreciprocated smiles can be interpreted as affective PE signals. In general, the predictive processing framework proposes that individuals aim to accurately predict the world by minimizing surprise. Experiencing too many PEs may thus become aversive and one possible strategy to avoid this may be to modify one’s behaviour to minimize PEs (K. Friston, 2005; K. J. Friston et al., 2014). We proposed that PE signals generated by a frowning counterpart could be minimized by disengaging from potentially frowning social agents, or by foregoing one’s own smile behaviour (Jeganathan & Breakspear, 2021).

We also found some evidence supporting the proposal that PE signals are fed back to motor areas. First, the SMC, a pre-motor area, was activated by the contrast of incongruent to congruent stimuli, despite these conditions being matched for participant action (smiling). Second, clinical participants with greater insular activity in response to incongruent stimuli had reduced smile amplitudes. Finally, previous work has shown that BOLD activity in the AI and inferior frontal gyrus are increased when one deliberately or spontaneously imitates positive facial expressions in others (Pohl et al., 2013). Our findings suggest that the same regions are activated when one’s positive facial expressions are followed by incongruent reactions from others, both in control and clinical cohorts. These seemingly paradoxical results can be reconciled within the predictive processing framework. In facial mimicry tasks, the initial mismatch between one’s own neutral face and the stimulus’ happy expression produces a PE signal in the AI and inferior frontal gyrus (consistent with our findings), which in turn motivates the participant to mimic the stimulus’ emotion to reduce the PE. This explanation requires an effective connection from visual regions to pre-motor areas, which is supported by our finding of the task induced affective PE signal increasing activity in the SMC. In sum, this convergent evidence suggests that unreciprocated smiles elicit PE signals that may be fed back to modify motor behaviour, potentially to reduce ongoing social behaviour.

In response to unreciprocated smiles, the clinical cohort displayed greater activations in left middle occipital gyrus and part of the left superior frontal gyrus that corresponds to dorsolateral prefrontal cortex, consistent with our hypothesis regarding greater prediction error signals in this cohort. This finding accords with the role of dorsolateral prefrontal cortex in inferring the intentions of others (van den Bos et al., 2011), threat vigilance, and consequent behavioural inhibition (Shackman et al., 2009). We next examined how brain responses varied with clinical differences within the clinical cohort. Notably, the left fusiform gyrus’ response to incongruent stimuli was greater in those with greater PANSS positive symptoms, potentially indicating greater attention to faces in individuals with more delusions and hallucinations. When smiles were not reciprocated, clinical participants with greater illness severity showed increased activity in the AI and SMC. Using DCM, these heightened neuronal responses were inferred to arise via greater disinhibition of the AI and SMC. These results align with recent models of schizophrenia indicating that dysfunctional NMDA receptors in inhibitory interneurons could lead to cortical disinhibition (Adams, 2019). Disengaging socially and avoiding smile behaviour are plausible strategies to minimize PEs. Therefore, increased sensitivity to unreciprocated smiles in those with severe psychotic illness, manifested as heightened PE signals, could lead to social withdrawal as a maladaptive strategy to avoid these PEs. This hypothesis is supported by the association between illness severity and reduced voluntary smile amplitude. While the task tested for participant-initiated smiling rather than reactionary smiling, in new reciprocal social interactions where back-and-forth smiling is expected, severely ill individuals may decrease their smiling to limit prediction errors arising from their conversational partner’s subsequent emotive responses. Moreover, clinical participants who exhibit affective blunting may have higher activation related to affective PEs because they have avoided these types of situations and thus these PE signals (Reed et al., 2020). In other words, affective PEs may be more salient for them compared to individuals who regularly engage in social interactions. PEs may also induce more anxiety in those who are socially withdrawn, leading to greater affective PE signals in this group. Thus, greater PEs and social withdrawal could persist by mutual reinforcement. Further longitudinal investigations could clarify the causal relationship between affective PEs and social withdrawal.

Overall, these results suggest a connection with the “PE hypothesis of schizophrenia”, which posits that symptoms in schizophrenia and schizoaffective disorder arise from disrupted predictive processing in the brain (Fletcher & Frith, 2009). To date, tests of this hypothesis have focussed predominantly on positive symptoms and neuropsychiatric features such as abnormal eye movements (Allen et al., 1990). Our study tests the extension of the PE hypothesis to the affective domain, suggesting that aberrant affective predictions contribute to affective exchanges and functional impairment.

Bilateral putamen, supramarginal gyrus, and early visual areas activated in response to the 2 × 2 interaction (Hh>Ha) > (Ah>Aa). These regions responded to congruency between participant action and stimulus emotion in conditions Hh and Aa, irrespective of whether the participant smiled or frowned. Supramarginal gyral activation in these conditions may reflect its role in distinguishing self-generated actions from observed actions (Macuga & Frey, 2011). Enhanced early visual activity in the congruent condition could reflect increased visual salience for emotionally congruent stimuli. In the 3-way interaction, bilateral postcentral gyri were activated by incongruent stimuli (Ha or Ah), particularly in the clinical group. We speculate that individuals with psychotic disorders may be more sensitive to emotional incongruence, leading to greater prediction error signals in facial proprioceptive areas.

Our findings should be interpreted in light of several caveats. First, our sample had a high proportion of males (85%), partly due to the greater incidence of schizophrenia in males (Aleman et al., 2003). This could have affected the results, as sex differences have been noted in emotional functioning and smiling (LaFrance et al., 2003). Groups were matched for sex ratio to reduce the impact of sex differences. Second, we used CGI as the primary outcome instead of PANSS. While PANSS can provide more fine-grained information on specific symptoms, changes in non-specific PANSS items such as uncooperativeness can markedly change PANSS total score while having a small impact on overall functioning (Wolfgang Fleischhacker, 2016). Conversely, a participant with only one or two major symptoms can have a low PANSS score but disproportionately high impairment. Consequently, we used the CGI as a well-validated and sensitive measure of severity and clinical impairment (Lader, 2000). The brain regions whose activity correlated with CGI did not overlap with the regions whose activity correlated with PANSS, supporting the relative independence of these measures. The choice of CGI was not pre-registered prior to the study being undertaken. Future studies may wish to pre-register the CGI as primary outcome to increase the robustness of the findings. Third, we used the (Ha > Hh) contrast to test for activity corresponding to incongruent feedback when an individual smiles. This contrast may not fully control for the general effects of viewing angry faces. To fully distinguish this effect of interest from the effect of angry faces, future work could include a null condition where the participant views angry or happy faces whilst remaining neutral themselves. Fourth, DCM has been subject to methodological critique (Lohmann et al., 2012). Concerns include the ‘third variable problem’ (unmeasured regions potentially influencing observed connectivity), the large parameter space, and Gaussian noise assumptions, although some of these concerns have been previously addressed (Breakspear, 2013; Kahan & Foltynie, 2013). We addressed these concerns using a minimal 3-node model, setting prior probabilities to zero, and constraining the search space with parametric empirical Bayes. Overall, our DCM results can be interpreted as model-based inferences about network dynamics rather than direct measurements of neural communication. Importantly, conventional fMRI analyses suggested that AI activation was associated with illness severity and smile amplitude in the clinical group, providing model-independent evidence for altered processing in severe psychotic illness. Next, 20% of the data was excluded due to problems with facial data or fMRI data. These exclusions could introduce selection bias, for example if participants with more severe symptoms were more likely to have poor data quality. However, there were no significant differences in illness severity between included and excluded participants. Finally, a potential limitation of the task is the use of voluntarily posed smiles rather than spontaneous expressions. This limits the task’s ecological validity. For instance, posed smile amplitude was not reduced in the clinical cohort. However, posed expressions ensured that participant smiles were repeated and accurately timed, allowing functional brain responses to be identified. Future studies can examine facial affect during real social interactions for increased ecological validity.

To conclude, we found that illness severity in schizophrenia and schizoaffective disorder is associated with enhanced disinhibition of the AI and SMC, leading to heightened affective PEs. Further work is needed to specifically test whether persons with psychotic disorders behave in a way to avoid these affective PE signals, such as disengaging from social encounters or expressing emotions. Affective PEs could be dampened by neuromodulatory treatments such as transcranial magnetic stimulation, or by psychological treatments such as exposure and response prevention to habituate participants to more salient PE signals, potentially counteracting the detrimental impact on patients’ functioning.

## Data Accessibility Statement

De-identified data can be shared subsequent to appropriate ethics approval and a data sharing agreement (due to patient privacy concerns).

## Additional File

The additional file for this article can be found as follows:

10.5334/cpsy.142.s1 Supplementary Materials. Supplementary Methods 1 to 8.
