# Supplementary material for: Illness Severity in Psychotic Disorders Amplifies Anterior Insula’s Sensitivity to Unreciprocated Smiles
Source: Comput Psychiatr. 2025 Dec 30;9(1):253–67. doi: 10.5334/cpsy.142 (PMC12758102; doi:10.5334/cpsy.142)
Supplement: Supplementary Materials. — Supplementary Methods 1 to 8. [file cpsy-9-1-142-s1.pdf]

Supplementary Materials

Supplementary Methods 1. Participant recruitment

All participants disavowed a previous history of traumatic brain injury, neurosurgery, neurological disorders, or current substance use disorder. Clinical participants were recruited from mental health services in the Hunter New England Local Health District in NSW, Australia. Consecutive eligible consumers of the mental health services were recruited during the recruitment period. Control participants were recruited using social media and billboard advertisements. Exclusion criteria for control participants included severe mental illnesses such as schizophrenia or bipolar disorder, previous hospital admission for any mental health condition, or having first-degree relatives with severe mental illnesses. Sex was ascertained based on clinical documentation. Sex and ethnicity were ascertained based on free text participant responses on a questionnaire. Participants provided written informed consent after receiving a complete description of the study. The study was approved by the Hunter New England Human Research Ethics Committee (2020/ETH00531).

Supplementary Methods 2. Face stimuli

Face stimuli were obtained from the Karolinska Directed Emotional Faces dataset (Lundqvist et al., 1998). Four different stimuli were used (two male, two female). All four stimulus faces occurred with equal probability across the 80 trials. Each stimulus face could react with neutral-to-happy or neutral-to-angry expression. Each reaction lasted 1750ms, comprising 30 frames presented at 20 frames per second, followed by the last frame (happy or angry expression) persisting for another 250ms. All stimuli were presented on a grey background at mean luminance. Face stimuli were set to greyscale and preprocessed using the SHINE toolbox in MATLAB (Willenbockel et al., 2010). Faces were luminance-matched to the screen background and set to half contrast. The background of each face stimulus image was matched to the screen background iteratively in terms of luminance histograms, and in terms of the rotational average of Fourier amplitude spectra.

Supplementary Methods 3. Video recording and stimulus presentation

Video footage was recorded with a Logitech c720 webcam (20 frames per second, resolution 800 x 600 pixels), and used to confirm adherence to task instructions. During the first run, stimuli were presented on a computer monitor (61cm diagonal, resolution 1920 x 1080 pixels) placed at eye level 50cm from the participant's eyes. During MR imaging, the BOLD 32 LCD monitor was placed 1.51m from the participant's eyes, and seen by the participant via a mirror in the MR headcoil. The monitor was calibrated with a GL Spectis 1.0 photometer and linearized in software, giving a mean and maximum luminance of 41.6 and 82.6 cd/m^2 respectively.

Supplementary Methods 4. Analysis of facial action unit time series

Full task time series (532s) was resampled to 20 frames per second and divided into trial-specific segments. Smile amplitude for each smile instruction trial was defined as the maximum activation of AU12 "Lip Corner Puller" during the trial.

While smiling is most closely associated with AU12 "Lip Corner Puller" and AU6 "Cheek Raiser", the precise mixture of AUs varies across participants. Data from all smile trials were concatenated. The first principal component (across AUs) of this data corresponded to the combination of AUs that most activated

in that participant during voluntary smiling. Similarly, the frown response was summarized using the first principal component of concatenated frown trials. AU time series were transformed to a single time series representing the first principal component, and re-synchronized such that time zero represented the trial's "trigger". Facial response onset and termination was estimated for each trial as follows.

The data was smoothed using a moving average filter with window length of either 250 ms or 450 ms, yielding two smoothed time series. Smoothing ensures that estimates of response onset and termination are able to capture larger and slower facial responses rather than small fast fluctuations such as micro-expressions. The mid-response time was defined as the time (milliseconds after trigger) when the 450 ms-smoothed time series achieved maximum slope (Supplementary Figure 1). In valid facial responses, the mid-response time was not too close (within 250 ms) of the start or end of the trial. Valid facial responses also had lower values at two timepoints before the mid-response (250 ms after trigger, and 250 ms before mid-response time) than at two timepoints after the mid-response (250 ms after mid-response time, and middle of the stimulus reaction phase). An initial estimate of response onset was obtained by finding the first local minimum in the 450 ms-smoothed time series to the left of the mid-response. The initial estimate usually overshot the actual response onset. The final estimate of response onset was obtained by finding the first local maximum in the 2nd derivative of the 250 ms-smoothed time series to the right of the initial estimate. An analogous procedure found initial and final estimates of response termination.

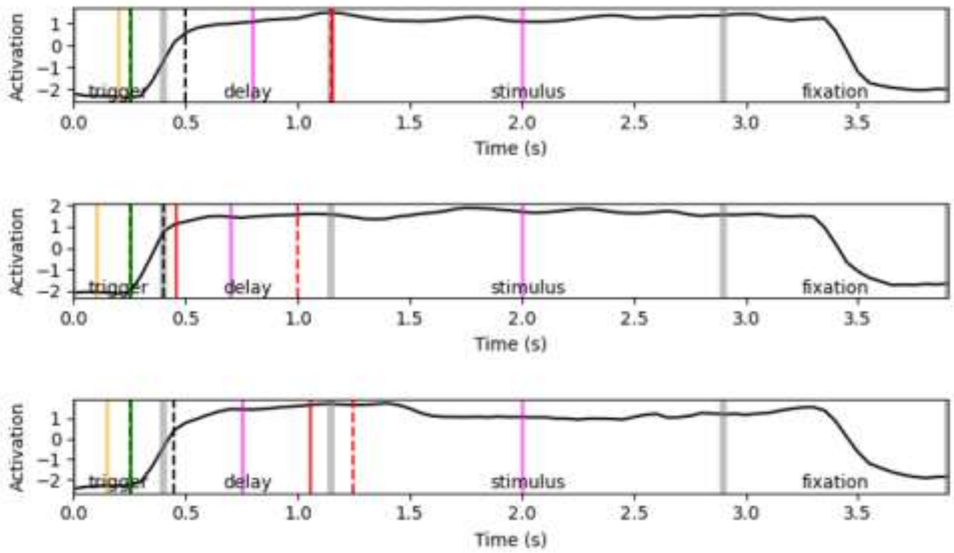

Supplementary Figure 1. Estimation of response onset and termination in single smile-instruction trials. Each plot represents a different trial in a single participant (participant 020). Black curve indicates the unsmoothed time series for the first principal component (across action units) of smile responses. Grey lines demarcate trial segments. Dashed black line indicates mid-response time. In valid facial responses, specific time points before the mid-response (yellow) must have smaller activation values than specific time points after the mid-response (magenta). Vertical lines also indicate initial estimate of response onset (dashed green), final estimate of response onset (solid green), initial estimate of response termination (dashed red), and final estimate of response termination (solid red).

Facial response latency was defined as the duration of time from the trigger to response onset. For each trial type (smile or frown instruction) in each participant, we calculated the median across trials of this trial-specific measure.

Supplementary Methods 5. MRI acquisition

Functional MRI data were acquired on a Siemens Magnetom Prisma 3T scanner with a 64-channel head and neck coil. The structural image comprised 208 T1-weighted MPRAGE images with 0.8 mm isotropic voxels (TR 2400 ms, TE 2.22 ms, flip angle 8°, field of view 256 mm). 665 functional volumes were collected during the Incongruent Facial Emotion task using a T2* weighted echo-planar imaging sequence with a multi-band acceleration of 6, comprising 60 slices with 2.4mm isotropic voxels (TR 800 ms, TE 35 ms, flip angle 53°, field of view 206 mm). The first five volumes were discarded to allow for magnetic saturation effects.

Supplementary Methods 6. Pre-processing with fMRIPrep, boilerplate text for a single exemplar participant

Results included in this manuscript come from preprocessing performed using *fMRIPrep* 23.0.0 (Esteban, Markiewicz, et al. (2018); Esteban, Blair, et al. (2018); RRID:SCR_016216), which is based on *Nipype* 1.8.5 (K. Gorgolewski et al. (2011); K. J. Gorgolewski et al. (2018); RRID:SCR_002502).

Preprocessing of B_0 inhomogeneity mappings
A total of 1 fieldmaps were found available within the input BIDS structure for this particular subject. A B_0-nonuniformity map (or *fieldmap*) was estimated based on two (or more) echo-planar imaging (EPI) references with topup (Andersson, Skare, and Ashburner (2003); FSL 6.0.5.1:57b01774).

Anatomical data preprocessing
A total of 1 T1-weighted (T1w) images were found within the input BIDS dataset.The T1-weighted (T1w) image was corrected for intensity non-uniformity (INU) with N4BiasFieldCorrection (Tustison et al. 2010), distributed with ANTs 2.3.3 (Avants et al. 2008, RRID:SCR_004757), and used as T1w-reference throughout the workflow. The T1w-reference was then skull-stripped with a *Nipype* implementation of the antsBrainExtraction.sh workflow (from ANTs), using OASIS30ANTs as target template. Brain tissue segmentation of cerebrospinal fluid (CSF), white-matter (WM) and gray-matter (GM) was performed on the brain-extracted T1w using fast (FSL 6.0.5.1:57b01774, RRID:SCR_002823, Zhang, Brady, and Smith 2001). Volume-based spatial normalization to two standard spaces (MNI152NLin6Asym, MNI152NLin2009cAsym) was performed through nonlinear registration with antsRegistration (ANTs 2.3.3), using brain-extracted versions of both T1w reference and the T1w template. The following templates were selected for spatial normalization: *FSL's MNI ICBM 152 non-linear 6th Generation Asymmetric Average Brain Stereotaxic Registration Model* [Evans et al. (2012), RRID:SCR_002823; TemplateFlow ID: MNI152NLin6Asym], *ICBM 152 Nonlinear Asymmetrical template version 2009c* [Fonov et al. (2009), RRID:SCR_008796; TemplateFlow ID: MNI152NLin2009cAsym].

Functional data preprocessing
For each of the 1 BOLD runs found per subject (across all tasks and sessions), the following preprocessing was performed. First, a reference volume and its skull-stripped version were generated by aligning and averaging 1 single-band references (SBRefs). Head-motion parameters with respect to the BOLD reference (transformation matrices, and six corresponding rotation and translation parameters) are estimated before any spatiotemporal filtering using mcflirt (FSL 6.0.5.1:57b01774, Jenkinson et al. 2002). The estimated *fieldmap* was then aligned with rigid-registration to the target EPI (echo-planar imaging) reference run. The field coefficients were mapped on to the reference EPI using the transform. BOLD runs were slice-time corrected to 0.351s (0.5 of slice acquisition range 0s-0.703s) using 3dTshift from AFNI (Cox and Hyde 1997, RRID:SCR_005927). The BOLD reference was then co-registered to the T1w reference using mri_coreg (FreeSurfer) followed by flirt (FSL 6.0.5.1:57b01774, Jenkinson and Smith 2001) with the boundary-based registration (Greve and Fischl 2009) cost-function. Co-registration was configured with six

degrees of freedom. First, a reference volume and its skull-stripped version were generated using a custom methodology of *fMRIPrep*. Several confounding time-series were calculated based on the *preprocessed BOLD*: framewise displacement (FD), DVARS and three region-wise global signals. FD was computed using two formulations following Power (absolute sum of relative motions, Power et al. (2014)) and Jenkinson (relative root mean square displacement between affines, Jenkinson et al. (2002)). FD and DVARS are calculated for each functional run, both using their implementations in *Nipype* (following the definitions by Power et al. 2014). The three global signals are extracted within the CSF, the WM, and the whole-brain masks. Additionally, a set of physiological regressors were extracted to allow for component-based noise correction (*CompCor*, Behzadi et al. 2007). Principal components are estimated after high-pass filtering the *preprocessed BOLD* time-series (using a discrete cosine filter with 128s cut-off) for the two *CompCor* variants: temporal (tCompCor) and anatomical (aCompCor). tCompCor components are then calculated from the top 2% variable voxels within the brain mask. For aCompCor, three probabilistic masks (CSF, WM and combined CSF+WM) are generated in anatomical space. The implementation differs from that of Behzadi et al. in that instead of eroding the masks by 2 pixels on BOLD space, a mask of pixels that likely contain a volume fraction of GM is subtracted from the aCompCor masks. This mask is obtained by thresholding the corresponding partial volume map at 0.05, and it ensures components are not extracted from voxels containing a minimal fraction of GM. Finally, these masks are resampled into BOLD space and binarized by thresholding at 0.99 (as in the original implementation). Components are also calculated separately within the WM and CSF masks. For each CompCor decomposition, the *k* components with the largest singular values are retained, such that the retained components' time series are sufficient to explain 50 percent of variance across the nuisance mask (CSF, WM, combined, or temporal). The remaining components are dropped from consideration. The head-motion estimates calculated in the correction step were also placed within the corresponding confounds file. The confound time series derived from head motion estimates and global signals were expanded with the inclusion of temporal derivatives and quadratic terms for each (Satterthwaite et al. 2013). Frames that exceeded a threshold of 0.5 mm FD or 1.5 standardized DVARS were annotated as motion outliers. Additional nuisance timeseries are calculated by means of principal components analysis of the signal found within a thin band (*crown*) of voxels around the edge of the brain, as proposed by (Patriat, Reynolds, and Birn 2017). The BOLD time-series were resampled into standard space, generating a *preprocessed BOLD run in MNI152NLin6Asym space*. First, a reference volume and its skull-stripped version were generated using a custom methodology of *fMRIPrep*. Automatic removal of motion artifacts using independent component analysis (ICA-AROMA, Pruim et al. 2015) was performed on the *preprocessed BOLD on MNI space* time-series after removal of non-steady state volumes and spatial smoothing with an isotropic, Gaussian kernel of 6mm FWHM (full-width half-maximum). Corresponding "non-aggresively" denoised runs were produced after such smoothing. Additionally, the "aggressive" noise-regressors were collected and placed in the corresponding confounds file. All resamplings can be performed with *a single interpolation step* by composing all the pertinent transformations (i.e. head-motion transform matrices, susceptibility distortion correction when available, and co-registrations to anatomical and output spaces). Gridded (volumetric) resamplings were performed using antsApplyTransforms (ANTs), configured with Lanczos interpolation to minimize the smoothing effects of other kernels (Lanczos 1964). Non-gridded (surface) resamplings were performed using mri_vol2surf (FreeSurfer).

Many internal operations of *fMRIPrep* use *Nilearn* 0.9.1 (Abraham et al. 2014, RRID:SCR_001362), mostly within the functional processing workflow.

References for fMRIPrep

Abraham, Alexandre, Fabian Pedregosa, Michael Eickenberg, Philippe Gervais, Andreas Mueller, Jean Kossaifi, Alexandre Gramfort, Bertrand Thirion, and Gael Varoquaux. 2014. “Machine Learning for Neuroimaging with Scikit-Learn.” *Frontiers in Neuroinformatics* 8. https://doi.org/10.3389/fninf.2014.00014.

Andersson, Jesper L. R., Stefan Skare, and John Ashburner. 2003. “How to Correct Susceptibility Distortions in Spin-Echo Echo-Planar Images: Application to Diffusion Tensor Imaging.” *NeuroImage* 20 (2): 870–88. https://doi.org/10.1016/S1053-8119(03)00336-7.

Avants, B. B., C. L. Epstein, M. Grossman, and J. C. Gee. 2008. “Symmetric Diffeomorphic Image Registration with Cross-Correlation: Evaluating Automated Labeling of Elderly and Neurodegenerative Brain.” *Medical Image Analysis* 12 (1): 26–41. https://doi.org/10.1016/j.media.2007.06.004.

Behzadi, Yashar, Khaled Restom, Joy Liau, and Thomas T. Liu. 2007. “A Component Based Noise Correction Method (CompCor) for BOLD and Perfusion Based fMRI.” *NeuroImage* 37 (1): 90–101. https://doi.org/10.1016/j.neuroimage.2007.04.042.

Cox, Robert W., and James S. Hyde. 1997. “Software Tools for Analysis and Visualization of fMRI Data.” *NMR in Biomedicine* 10 (4-5): 171–78. https://doi.org/10.1002/(SICI)1099-1492(199706/08)10:4/5<171::AID-NBM453>3.0.CO;2-L.

Esteban, Oscar, Ross Blair, Christopher J. Markiewicz, Shoshana L. Berleant, Craig Moodie, Feilong Ma, Ayse Ilkay Isik, et al. 2018. “fMRIPrep 23.0.0.” *Software*. https://doi.org/10.5281/zenodo.852659.

Esteban, Oscar, Christopher Markiewicz, Ross W Blair, Craig Moodie, Ayse Ilkay Isik, Asier Erramuzpe Aliaga, James Kent, et al. 2018. “fMRIPrep: A Robust Preprocessing Pipeline for Functional MRI.” *Nature Methods*. https://doi.org/10.1038/s41592-018-0235-4.

Evans, AC, AL Janke, DL Collins, and S Baillet. 2012. “Brain Templates and Atlases.” *NeuroImage* 62 (2): 911–22. https://doi.org/10.1016/j.neuroimage.2012.01.024.

Fonov, VS, AC Evans, RC McKinstry, CR Almli, and DL Collins. 2009. “Unbiased Nonlinear Average Age-Appropriate Brain Templates from Birth to Adulthood.” *NeuroImage* 47, Supplement 1: S102. https://doi.org/10.1016/S1053-8119(09)70884-5.

Gorgolewski, K., C. D. Burns, C. Madison, D. Clark, Y. O. Halchenko, M. L. Waskom, and S. Ghosh. 2011. “Nipype: A Flexible, Lightweight and Extensible Neuroimaging Data Processing Framework in Python.” *Frontiers in Neuroinformatics* 5: 13. https://doi.org/10.3389/fninf.2011.00013.

Gorgolewski, Krzysztof J., Oscar Esteban, Christopher J. Markiewicz, Erik Ziegler, David Gage Ellis, Michael Philipp Notter, Dorota Jarecka, et al. 2018. “Nipype.” *Software*. https://doi.org/10.5281/zenodo.596855.

Greve, Douglas N, and Bruce Fischl. 2009. “Accurate and Robust Brain Image Alignment Using Boundary-Based Registration.” *NeuroImage* 48 (1): 63–72. https://doi.org/10.1016/j.neuroimage.2009.06.060.

Jenkinson, Mark, Peter Bannister, Michael Brady, and Stephen Smith. 2002. “Improved Optimization for the Robust and Accurate Linear Registration and Motion Correction of Brain Images.” *NeuroImage* 17 (2): 825–41. https://doi.org/10.1006/nimg.2002.1132.

Jenkinson, Mark, and Stephen Smith. 2001. “A Global Optimisation Method for Robust Affine Registration of Brain Images.” *Medical Image Analysis* 5 (2): 143–56. https://doi.org/10.1016/S1361-8415(01)00036-6.

Lanczos, C. 1964. “Evaluation of Noisy Data.” *Journal of the Society for Industrial and Applied Mathematics Series B Numerical Analysis* 1 (1): 76–85. https://doi.org/10.1137/0701007.

Patriat, Rémi, Richard C. Reynolds, and Rasmus M. Birn. 2017. “An Improved Model of Motion-Related Signal Changes in fMRI.” *NeuroImage* 144, Part A (January): 74–82. https://doi.org/10.1016/j.neuroimage.2016.08.051.

Power, Jonathan D., Anish Mitra, Timothy O. Laumann, Abraham Z. Snyder, Bradley L. Schlaggar, and Steven E. Petersen. 2014. "Methods to Detect, Characterize, and Remove Motion Artifact in Resting State fMRI." *NeuroImage* 84 (Supplement C): 320–41. https://doi.org/10.1016/j.neuroimage.2013.08.048.
Pruim, Raimon H. R., Maarten Mennes, Daan van Rooij, Alberto Llera, Jan K. Buitelaar, and Christian F. Beckmann. 2015. "ICA-AROMA: A Robust ICA-Based Strategy for Removing Motion Artifacts from fMRI Data." *NeuroImage* 112 (Supplement C): 267–77. https://doi.org/10.1016/j.neuroimage.2015.02.064.
Satterthwaite, Theodore D., Mark A. Elliott, Raphael T. Gerraty, Kosha Ruparel, James Loughead, Monica E. Calkins, Simon B. Eickhoff, et al. 2013. "An improved framework for confound regression and filtering for control of motion artifact in the preprocessing of resting-state functional connectivity data." *NeuroImage* 64 (1): 240–56. https://doi.org/10.1016/j.neuroimage.2012.08.052.
Tustison, N. J., B. B. Avants, P. A. Cook, Y. Zheng, A. Egan, P. A. Yushkevich, and J. C. Gee. 2010. "N4itk: Improved N3 Bias Correction." *IEEE Transactions on Medical Imaging* 29 (6): 1310–20. https://doi.org/10.1109/TMI.2010.2046908.
Zhang, Y., M. Brady, and S. Smith. 2001. "Segmentation of Brain MR Images Through a Hidden Markov Random Field Model and the Expectation-Maximization Algorithm." *IEEE Transactions on Medical Imaging* 20 (1): 45–57. https://doi.org/10.1109/42.906424.

Supplementary Methods 7. fMRI general linear model

The general linear model was implemented in SPM version 12 v7771 (K. J. Friston et al., 2006). Within single participants, regressors of interest comprised the 4 task conditions: *Hh, Ha, Ah, Aa*. Each task regressor was modelled as a box function with value 1 during the stimulus reaction (duration 1750 ms) and 0 elsewhere. Task regressors aimed to measure neural responses to producing a facial expression and viewing the stimulus face. Task regressors had value 0 during the trigger, delay and fixation phases, as participants' facial movements occurred during these phases. This reduced the contribution of movement artefact. Additional regressors corresponding to instruction (smile or frown) were also included (duration 500 ms), but neural responses to seeing the instruction were not the focus of this study. Regressors were convolved with a prototypic hemodynamic response function. Nuisance covariates were included in the design matrix. This comprised the CSF, white matter, movement, and physiological regressors. 24 movement regressors were included, comprising translations and rotations in all 3 axes, their first derivatives, squared regressors, and squared derivatives. Physiological regressors were obtained by processing pulse photoplethysmogram (PPG) and respiratory belt recordings with Matlab PhysIO Toolbox version 5.1.2 (Frässle et al., 2021; Kasper et al., 2017).

Effects at each voxel were estimated with a least-squares algorithm. Regionally specific effects were estimated with linear t-contrasts. *Hh* and *Aa* trials were termed "congruent" while *Ha* and *Ah* trials were termed "incongruent". The full interaction contrast (incongruent minus congruent stimulus) used contrast [-1,1,1,-1]. The main effect of participant action (smile minus frown) used contrast [1,1,-1,-1]. The main effect of stimulus (happy minus angry) used contrast [1,-1,1,-1]. The participant's response to an incongruent stimulus after smiling was modelled with contrast [-1,1]. The single-participant t-contrast images were combined in a second level random effects analysis across the entire cohort. Cohort level activations for each contrast were identified with one-sample t tests. Group differences were assessed with two-sample t tests. Correspondence between continuous variables (such as clinical measures) and fMRI activations were tested by including the continuous variable as a regressor in the second level design matrix. A height threshold of $p = 0.005$ and cluster extent threshold corresponding to $p = 0.05$ (FDR-corrected) were used (K. J. Friston et al., 2006). This method provides adequate false positive control with conservative thresholds

and moderate cluster sizes (Cox et al., 2017; Flandin & Friston, 2019). All significant clusters exceeded 100 voxels, well above the liberal thresholds previously identified as problematic (Eklund et al., 2016).

Supplementary Methods 8. DCM method, masks, and regressors

DCM is a modelling framework for effective connectivity, which is based on the principles of dynamic systems theory (K. J. Friston et al., 2003, 2019). DCM for fMRI has been validated on simulated data (Razi et al., 2015), empirical data using invasive EEG recordings (David et al., 2008), and comparison to known anatomical connectivity (Stephan et al., 2009). DCM instantiates a parsimonious model of distributed brain activity, incorporating unknown direct and indirect anatomical connections into effective connectivity. Effective connectivity is sometimes associated with underlying anatomical connections (Stephan et al., 2009), but this is not necessary (K. J. Friston et al., 2003). Effective connections in DCM are often mediated by poly-synaptic pathways (Daunizeau et al., 2011). For example, effective connectivity between FUS and AI could be instantiated by anatomical connectivity between FUS and an unspecified third region, and a further connection between AI and that same region.

Overfitting is a potential issue in all generative and dynamic models of neuroimaging data, due to the large number of possible models and parameters (Valdes-Sosa et al., 2011). We used a 3-node model to limit the search space and prevent combinatorial explosion. We selected these regions based on a priori hypotheses from the emotion processing literature (Benuzzi et al., 2023; Paulus & Stein, 2006). The right fusiform cortex serves as the visual input node, while anterior insula and supplementary motor cortex were identified in our whole-brain analysis as responsive to unreciprocated smiles. Unmeasured regions (e.g., amygdala, prefrontal cortex) likely contribute to this network and could be examined in future studies with more complex models. To further reduce the risk of overfitting, prior probabilities of connectivity strengths were set to zero, so that effective connections would only be inferred if there was sufficiently strong evidence in the data (Stephan et al., 2009).

To further reduce the risk of overfitting, models were adjudicated using the free energy criterion which measures model accuracy while penalizing complex models (K. J. Friston & Stephan, 2007; Penny, 2012).. Model selection was conducted at the group level. To effectively explore the model space, we used parametric empirical Bayes (PEB) with Bayesian Model Reduction (BMR) (K. J. Friston et al., 2016) to iteratively prune parameters that did not contribute to model evidence, identifying the modulatory parameters that (i) explained the common effect of unreciprocated smiles in all participants, and (ii) individual differences in illness severity. Bayesian model averaging computed the weighted average parameter values from the best reduced models. PEB inverts one full DCM per participant. Group-level parameters inform participant-level parameter estimation, further constraining the search space (Zeidman et al., 2019). All reported parameters exceeded a cutoff posterior probability of 0.95, indicating *strong evidence* for the parameter's influence on the model (Zeidman et al., 2019).

Group-level masks were generated for the incongruent stimulus, and for the overall signal during the stimulus reaction phase, with a height threshold of $p = 0.05$ and cluster extent threshold corresponding to $p = 0.05$ (FDR-corrected). Three volumes of interest were generated: right fusiform (FUS, 1607 voxels), right anterior insula (AI, 362 voxels), and right supplementary motor cortex (SMC, 821 voxels). The FUS volume was defined as the conjunction of the overall response mask and the right fusiform region in the AAL template(Rolls et al., 2020). The AI volume was defined as the conjunction of the incongruent stimulus mask and the right insula region in the AAL template. The SMC volume was defined as the cluster within the incongruent stimulus mask corresponding to the right supplementary motor cortex. The regressors

corresponding to smile-instruction trials (*Ha* and *Hh*) were re-parameterized to a smile regressor (*Ha* + *Hh*) and an incongruent stimulus regressor (*Ha* – *Hh*). The smile regressor corresponded not only to the participant smiling, but also to the participant simultaneously visualizing the stimulus' facial expression.

Supplementary Table 1. Group means for demographics variables

	**Clinical cohort**	**Healthy controls**	**Group difference**
n	32	28	
Age, mean years (SD)	36.5 (8.3)	43.3 (6.4)	$U = 222, p < 0.001$
Sex male, n (%)	27 (84.4%)	24 (85.7%)	$\chi^2 = 0.000, p = 1.000$
Education, mean years (SD)	11.9 (1.9)	12.1 (2.8)	$U = 473.5, p = 0.691$
IQ, mean (SD)	101.7 (11.8)	109.6 (11.9)	$t(58) = -2.577; p = 0.013$

Supplementary Table 2. Mean and standard deviation of clinical rating scales for the clinical cohort.

**Scale**	**Mean**	**Standard deviation**
Clinical Global Impression (CGI)	3.56	1.29
Social and Occupational Functioning Scale (SOFAS)	61.3	16.6
PANSS positive symptoms	12.4	4.59
PANSS negative symptoms	17.0	5.73
PANSS general psychopathology	27.4	5.52
Hamilton Depression Rating Scale (HAM-D)	3.00	3.40
Simpson Angus Scale (SAS)	1.19	1.60

Supplementary Table 3. Significant fMRI clusters in the Incongruent Facial Emotion task: main effects and interaction across all participants

**Condition**	**Cluster region**	**Cluster size**	**Cluster-level *p* (FDR-corrected)**	**Peak coordinates (x, y, z)**
Overall effect of task, F-contrast	Visual areas, fusiform gyrus, pre-central gyrus, post-central gyrus, parietal association areas	77030	<0.001	32, -66, -14
	R superior temporal	215	<0.001	68, -22, 6
	R superior frontal	892	<0.001	24, 48, 12
	L putamen	101	0.017	-20, 22, 0
	R transverse temporal	100	0.017	46, -14, 2
	Several other clusters			
Main effect of participant action, F-contrast	Precuneus	1496	<0.001	0, -62, 54
	L occipital fusiform	232	<0.001	-22, -90, -14
	L posterior orbital	1081	<0.001	-30, 28, -18
	R cerebellum	195	<0.001	8, -46, -46
	L lingual	653	<0.001	-12, 52, -2
	Several other clusters including visual areas, inferior temporal, superior parietal lobule			
Main effect of stimulus emotion, F-contrast	R fusiform, R inferior temporal, R parahippocampal	1726	<0.001	38, -44, -22
	R lingual	238	<0.001	14, -80, -12
	L fusiform	227	<0.001	-42, -42, -16
	L lingual	865	<0.001	0, -74, 2
	R precentral	309	<0.001	58, 14, 24
	Several other clusters			
Interaction:	R putamen	919	<0.001	30, -14, 6

Congruent>Incongruent ((Hh>Ha)>(Ah>Aa))				
	R visual, R fusiform, R angular, R superior parietal	3948	<0.001	50, -56, -14
	L putamen	560	<0.001	-20, 0, -8
	L occipital pole	986	<0.001	-10, -102, 6
	L angular gyrus	379	<0.001	-36, -74, 32
	R cerebellum	143	0.049	40, -64, -38
Interaction: Incongruent>Congruent ((Hh>Ha)<(Ah>Aa))	None			

Supplementary Table 4. Significant fMRI clusters for incongruent stimuli after smiling (Ha>Ha contrast)

**Cluster region**	**Cluster size**	**Cluster-level *p* (FDR-corrected)**	**Peak coordinates (x, y, z)**
R anterior insula, R inferior frontal gyrus	365	< 0.001	34, 20, 2
R supplementary motor cortex	167	0.025	12, 6, 52
L anterior insula	153	0.026	-42, 16, -8

Supplementary Table 5. Significant fMRI clusters in the Incongruent Facial Emotion task: clinical group interactions

**Condition**	**Cluster region**	**Cluster size**	**Cluster-level *p* (FDR-corrected)**	**Peak coordinates (x, y, z)**
Group*Interaction (healthy>clinical)*(incongruent>congruent)	None			
Group*Interaction (clinical>healthy)*(incongruent>congruent)	R postcentral gyrus	181	0.001	64,-8,20
	L postcentral gyrus			
		260	<0.001	-60,-10,34
Group*Incongruent after smiling (healthy>clinical)*(Ha>Hh)	None			
Group*Incongruent after smiling (clinical>healthy)*(Ha>Hh)	L middle occipital gyrus	167	0.001	-44,-78,34
	L superior frontal gyrus			
		171	0.001	-24,26,44
Greater illness severity * (Ha>Hh) in clinical group	R cerebellum	399	<0.001	8, -60, -54
	R ventral midbrain	1125	<0.001	18, -18, -14
	L middle frontal gyrus	2973	<0.001	-40, 6, 38
	R precuneus	1332	<0.001	6, -62, 38
	R anterior insula	189	0.002	42, -2, 4
	L supplementary motor cortex	205	0.002	-8, 6, 56
	Several other clusters			
Lesser illness severity * (Ha>Hh) in clinical group	None			
Greater smile amplitude * (Ha>Hh) in clinical group	None			
Lesser smile amplitude * (Ha>Hh) in clinical group	R amygdala	149	0.021	26, -2, -16
	R anterior insula, R central operculum	633	<0.001	62, -6, 14
	L postcentral gyrus	328	<0.001	-58, -12, 24
	R middle temporal gyrus	365	<0.001	56, -52, 12

Greater PANSS positive scale * (Ha>Hh) in clinical group	Cerebellar vermis	417	<0.001	2,-72,-38
	L angular gyrus	221	0.013	-34,-52,36
	L medial precentral gyrus	199	0.016	-4,-22,60
	L fusiform gyrus	174	0.026	-36,-48,-22
Lesser PANSS positive scale * (Ha>Hh) in clinical group	None			
Greater PANSS negative scale * (Ha>Hh) in clinical group	None			
Lesser PANSS negative scale * (Ha>Hh) in clinical group	None			
Greater antipsychotic dose * (Ha>Hh) in clinical group	Right precentral gyrus	210	<0.001	58,6,24
Lesser antipsychotic dose * (Ha>Hh) in clinical group	None			

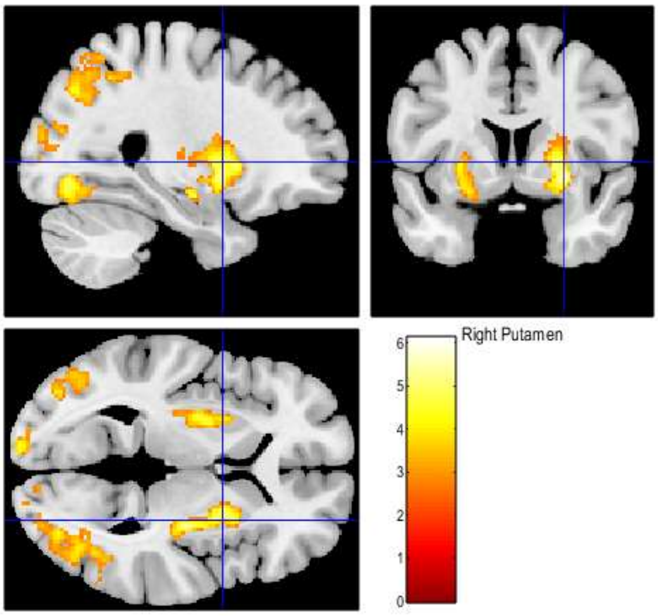

Supplementary Figure 2. Clusters with significant activation for the contrast Congruent>Incongruent, across smile instruction and frown instruction trials. MNI coordinates (25.2, 3.4, 4.0)

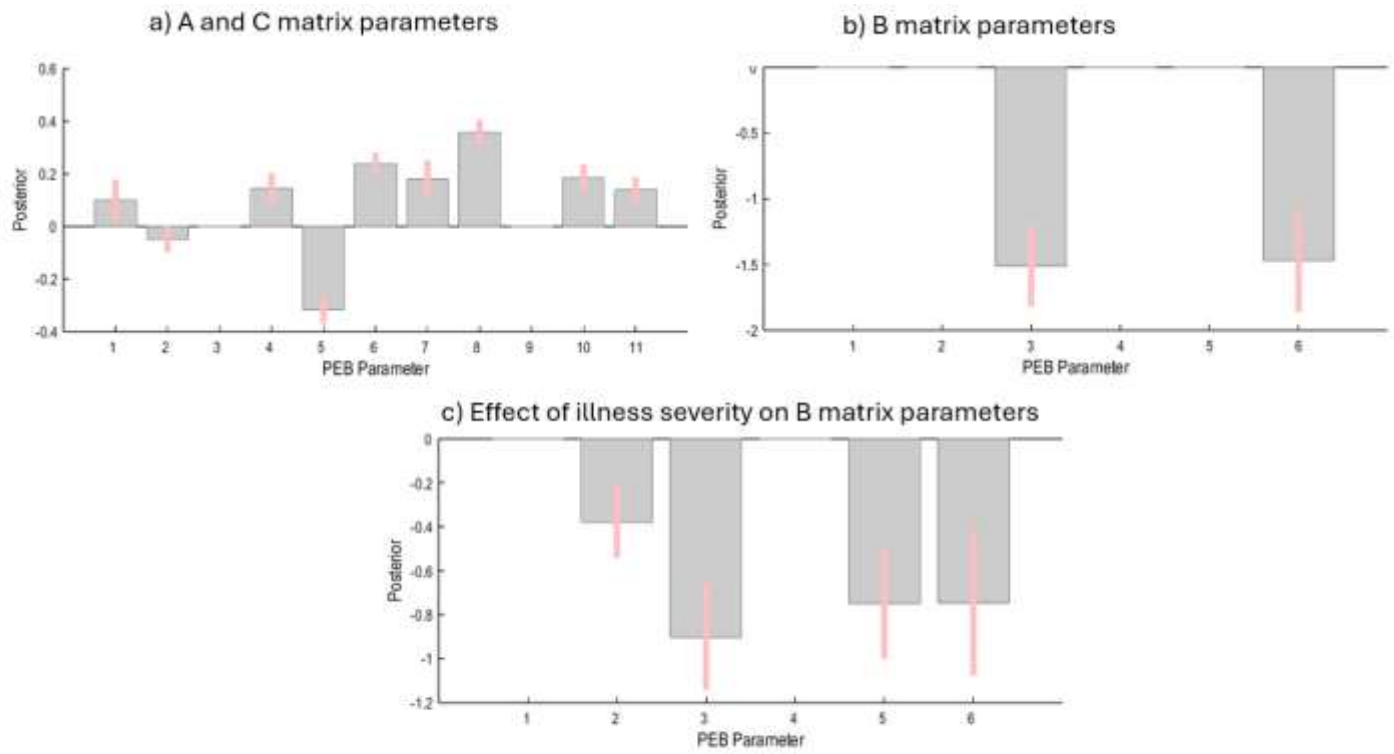

Supplementary Figure 3. Expected posterior and 95% Bayesian credible interval for bilinear modulatory parameters in Parametric empirical Bayes. Numerical labels on the horizontal axis indicate the index of each parameter. Plot (a) represents estimates for A-matrix followed by C-matrix parameters. In plot (a), PEB parameters from left to right are (1) A-matrix connectivity from FUS to FUS, (2) FUS to RAI, (3) FUS to RSMC, (4) RAI to FUS, (5) RAI to RAI, (6) RAI to RSMC, (7) RSMC to FUS, (8) RSMC to RAI, (9) RSMC to RSMC, (10) C-matrix effect of smile action on FUS, and (11) effect of frown action on FUS. Plot (b) represents B-matrix parameters. Plot (c) represents the modulation of B-matrix parameters by illness severity. In plots (b) and (c), PEB parameters from left to right are effect of smile connectivity on (1) connectivity from FUS to AI, (2) connectivity from FUS to SMC, effect of incongruent stimuli on (3) AI self-connectivity, (4) connectivity from AI to SMC, (5) connectivity from SMC to AI, and (6) SMC self-connectivity. All surviving parameters had posterior probability > 0.95 indicating “strong evidence”

References

Cox, R. W., Chen, G., Glen, D. R., Reynolds, R. C., & Taylor, P. A. (2017). fMRI clustering and false-positive rates. *Proceedings of the National Academy of Sciences*, *114*(17), E3370–E3371. https://doi.org/10.1073/pnas.1614961114

Daunizeau, J., David, O., & Stephan, K. E. (2011). Dynamic causal modelling: A critical review of the biophysical and statistical foundations. *NeuroImage*, *58*(2), 312–322. https://doi.org/10.1016/j.neuroimage.2009.11.062

David, O., Guillemain, I., Saillet, S., Reyt, S., Deransart, C., Segebarth, C., & Depaulis, A. (2008). Identifying Neural Drivers with Functional MRI: An Electrophysiological Validation. *PLOS Biology*, *6*(12), e315. https://doi.org/10.1371/journal.pbio.0060315

Eklund, A., Nichols, T. E., & Knutsson, H. (2016). Cluster failure: Why fMRI inferences for spatial extent have inflated false-positive rates. *Proceedings of the National Academy of Sciences*, *113*(28), 7900–7905. https://doi.org/10.1073/pnas.1602413113

Flandin, G., & Friston, K. J. (2019). Analysis of family-wise error rates in statistical parametric mapping using random field theory. *Human Brain Mapping*, *40*(7), 2052–2054. https://doi.org/10.1002/hbm.23839

Frässle, S., Aponte, E. A., Bollmann, S., Brodersen, K. H., Do, C. T., Harrison, O. K., Harrison, S. J., Heinzle, J., Iglesias, S., Kasper, L., Lomakina, E. I., Mathys, C., Müller-Schrader, M., Pereira, I., Petzschner, F. H., Raman, S., Schöbi, D., Toussaint, B., Weber, L. A., … Stephan, K. E. (2021). TAPAS: An Open-Source Software Package for Translational Neuromodeling and Computational Psychiatry. *Frontiers in Psychiatry*, *12*, 680811. https://doi.org/10.3389/fpsyt.2021.680811

Friston, K. J., Ashburner, J., Kiebel, S. J., Nichols, T. E., & Penny, W. D. (2006). *Statistical Parametric Mapping: The Analysis of Functional Brain Images*. Academic Press. https://shop.elsevier.com/books/statistical-parametric-mapping-the-analysis-of-functional-brain-images/penny/978-0-12-372560-8

Friston, K. J., Litvak, V., Oswal, A., Razi, A., Stephan, K. E., van Wijk, B. C. M., Ziegler, G., & Zeidman, P. (2016). Bayesian model reduction and empirical Bayes for group (DCM) studies. *NeuroImage*, *128*, 413–431. https://doi.org/10.1016/j.neuroimage.2015.11.015

Friston, K. J., Preller, K. H., Mathys, C., Cagnan, H., Heinzle, J., Razi, A., & Zeidman, P. (2019). Dynamic causal modelling revisited. *NeuroImage*, *199*, 730–744. https://doi.org/10.1016/j.neuroimage.2017.02.045

Friston, K. J., & Stephan, K. E. (2007). Free-energy and the brain. *Synthese*, *159*(3), 417–458. https://doi.org/10.1007/s11229-007-9237-y

Kasper, L., Bollmann, S., Diaconescu, A. O., Hutton, C., Heinzle, J., Iglesias, S., Hauser, T. U., Sebold, M., Manjaly, Z.-M., Pruessmann, K. P., & Stephan, K. E. (2017). The PhysIO Toolbox for Modeling Physiological Noise in fMRI Data. *Journal of Neuroscience Methods*, *276*, 56–72. https://doi.org/10.1016/j.jneumeth.2016.10.019

Penny, W. D. (2012). Comparing Dynamic Causal Models using AIC, BIC and Free Energy. *NeuroImage*, *59*(1), 319–330. https://doi.org/10.1016/j.neuroimage.2011.07.039

Razi, A., Kahan, J., Rees, G., & Friston, K. J. (2015). Construct validation of a DCM for resting state fMRI. *NeuroImage*, *106*, 1–14. https://doi.org/10.1016/j.neuroimage.2014.11.027

Rolls, E. T., Huang, C.-C., Lin, C.-P., Feng, J., & Joliot, M. (2020). Automated anatomical labelling atlas 3. *NeuroImage*, *206*, 116189. https://doi.org/10.1016/j.neuroimage.2019.116189

Stephan, K. E., Tittgemeyer, M., Knösche, T. R., Moran, R. J., & Friston, K. J. (2009). Tractography-based priors for dynamic causal models. *NeuroImage*, *47*(4), 1628–1638. https://doi.org/10.1016/j.neuroimage.2009.05.096

Valdes-Sosa, P. A., Roebroeck, A., Daunizeau, J., & Friston, K. (2011). Effective connectivity: Influence, causality and biophysical modeling. *NeuroImage*, *58*(2), 339–361. https://doi.org/10.1016/j.neuroimage.2011.03.058

Willenbockel, V., Sadr, J., Fiset, D., Horne, G., Gosselin, F., & Tanaka, J. (2010). The SHINE toolbox for controlling low-level image properties. *Journal of Vision*, *10*(7), 653. https://doi.org/10.1167/10.7.653

Zeidman, P., Jafarian, A., Seghier, M. L., Litvak, V., Cagnan, H., Price, C. J., & Friston, K. J. (2019). A guide to group effective connectivity analysis, part 2: Second level analysis with PEB. *Neuroimage*, *200*, 12–25. https://doi.org/10.1016/j.neuroimage.2019.06.032
